# Precision drugging of the MAPK pathway in head and neck cancer

**DOI:** 10.1038/s41525-022-00293-1

**Published:** 2022-03-16

**Authors:** Hoi-Lam Ngan, Chun-Ho Law, Yannie Chung Yan Choi, Jenny Yu-Sum Chan, Vivian Wai Yan Lui

**Affiliations:** 1 School of Biomedical Sciences, Faculty of Medicine, The Chinese University of Hong Kong, Hong Kong SAR, Hong Kong; 2 Faculty of Medicine, The Chinese University of Hong Kong, Hong Kong SAR, Hong Kong; 3 Georgia Cancer Center, and Department of Medicine, Medical College of Georgia, Augusta University, Georgia, GA 30912 USA

**Keywords:** Drug development, Cancer genomics, Head and neck cancer, Translational research

## Abstract

The mitogen-activating protein kinase (MAPK) pathway is central for cell proliferation, differentiation, and senescence. In human, germline defects of the pathway contribute to developmental and congenital head and neck disorders. Nearly 1/5 of head and neck squamous cell carcinoma (HNSCC) harbors MAPK pathway mutations, which are largely activating mutations. Yet, previous approaches targeting the MAPK pathway in HNSCC were futile. Most recent clinical evidences reveal remarkable, or even exceptional pharmacologic vulnerabilities of *MAPK1*-mutated, *HRAS-*mutated, *KRAS-*germline altered, *as well as BRAF-*mutated HNSCC patients with various targeted therapies, uncovering diverse opportunities for precision drugging this pathway at multiple “genetically condemned” nodes. Further, recent patient tumor omics unveil novel effects of MAPK aberrations on direct induction of CD8^+^ T cell recruitment into the HNSCC microenvironment, providing evidences for future investigation of precision immunotherapy for this large subset of patients. MAPK pathway-mutated HNSCC should warrant precision therapy assessments in vigorous manners.

## Introduction

The mitogen-activated protein kinase (MAPK) pathway is a key signaling hub integrating extracellular signals for the control of cell proliferation, survival, differentiation, senescence as well as drug resistance^1,2^. This pathway comprises an array of kinases, including the RAFs (*A-/B-/C-RAF*), RASs (*H/K/NRAS*), MEKs (*MEK1/2*), MAPKs [*MAPK1 (ERK2), MAPK3 (ERK1)*], adaptor molecules (*GRB2, SHC1/2/3/4*), and ERK1/2-specific negative regulators called the dual-specificity phosphatases (*DUSP3/5/6/7/9*). Constitutive activation of key kinases, such as BRAF, HRAS, KRAS, and MAPK1/3, are well-known to drive human oncogenesis via transcriptional activation, cross-talks with other oncogenic pathways (e.g., PI3K and JAK/STAT pathways), and recently, via immuno-modulatory activities as reported in some cancers^3–7^.

**Fig. 1:**
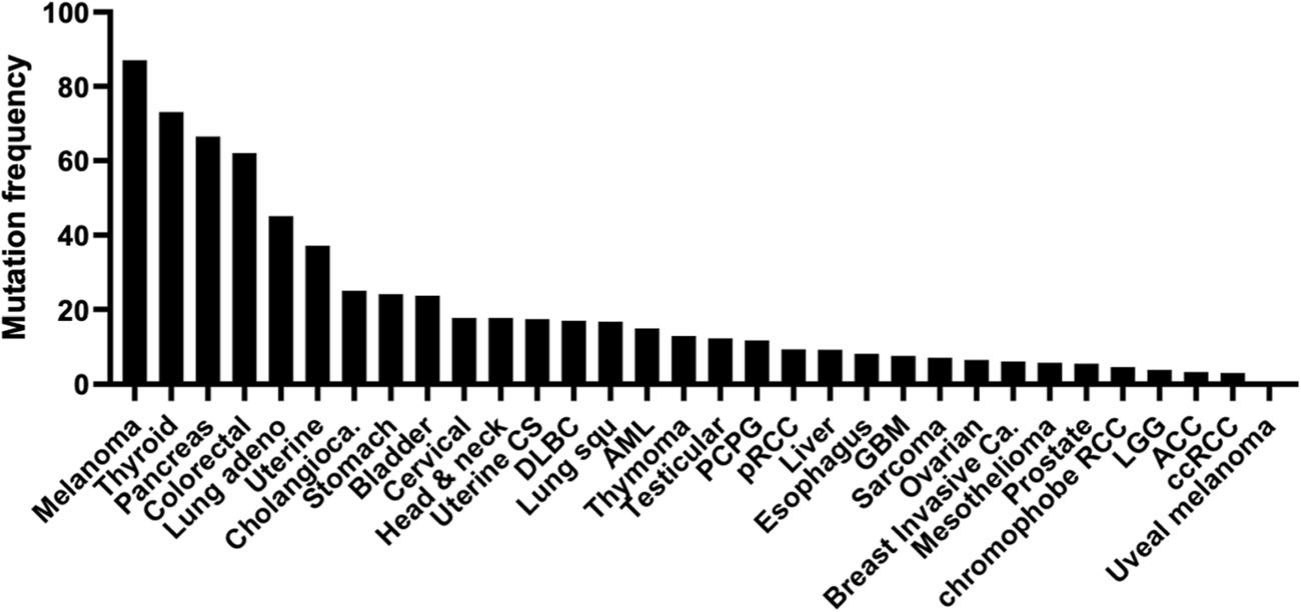
Over 30 human cancer types characterized by the Cancer Genome Atlas (TCGA, USA) harbor noticeable subsets of patients bearing somatic mutations of the MAPK pathway. *Abbreviation:* Melanoma Skin Cutaneous Melanoma, Thyroid Thyroid Carcinoma, Pancreas Pancreatic Adenocarcinoma, Colorectal Colorectal Adenocarcinoma, Lung adeno Lung Adenocarcinoma, Uterine Uterine Corpus Endometrial Carcinoma, Cholangiocarcinoma Cholangiocarcinoma, Stomach Stomach Adenocarcinoma, Bladder Bladder Urothelial Carcinoma, Cervical Cervical Squamous Cell Carcinoma & Endocervical Adenocarcinoma, Head & neck Head and Neck Squamous Cell Carcinoma, Uterine CS Uterine Carcinosarcoma, DLBC Lymphoid Neoplasm Diffuse Large B-cell Lymphoma, Lung squ Lung Squamous Cell Carcinoma, AML Acute Myeloid Leukemia, Thymoma Thymoma, Testicular Testicular Germ Cell Tumors, PCPG Pheochromocytoma and Paraganglioma, pRCC Kidney Renal Papillary Cell Carcinoma, Liver Liver Hepatocellular Carcinoma, Esophagus Esophageal Adenocarcinoma, GBM Glioblastoma Multiforme, Sarcoma Sarcoma, Ovarian Ovarian Serous Cystadenocarcinoma, Breast Invasive Ca. Breast Invasive Carcinoma, Mesothelioma Mesothelioma, Prostate Prostate Adenocarcinoma, chromophobe RCC Kidney Chromophobe, LGG Brain Lower Grade Glioma, ACC Adrenocortical Carcinoma, ccRCC Kidney Renal Clear Cell Carcinoma, Uveal Melanoma Uveal Melanoma.

In addition to external growth stimuli, somatic mutations of this pathway can cause robust constitutive MAPK activation in various solid tumors. As of today, two MAPK-driven cancers are known to be predominantly affected by somatic *BRAF* p.V600E activating mutations (~50% cases mutated in melanoma and thyroid cancer)^8,9^. Additional 30 cancer types also harbor noticeable subsets of MAPK pathway-mutated patients (2.99-83.4% cases) (Fig. 1). Many of the MAPK pathway mutations, though not all, have been demonstrated to be oncogenic in nature, and potentially druggable as well. These include *KRAS* p.G12C, *MAP2K1* p.Q56P, and *MAPK1* p.D321N, etc^10–12^. Prior to the era of genomic medicine, non-precision drugging (i.e., non-mutation based) of the MAPK pathway with many MAPK pathway inhibitors had miserably failed in clinical trials for almost all cancers. Yet, advances in precision drugging of the *BRAF* p.V600E mutation alone with BRAF inhibitors have now extended the survival of numerous melanoma, thyroid, and non-small cell lung cancer (NSCLC) patients worldwide^13^. However, for most other cancers, MAPK pathway has remained clinically undruggable till now despite common occurrences of MAPK mutations across all cancers.

### Genetic aberrations of MAPK pathway in head and neck syndromes, and cancer

Head and neck squamous cell carcinoma (HNSCC) is a highly aggressive cancer arising from the epithelial lining of the head and neck region. It has a rising global incidence of >0.83 million new cases/year (2018, International Agency for Research on Cancer, IARC^14^). Diverse etiologies contribute to HNSCC carcinogenesis, including exposures to carcinogens, including tobacco, alcohol, betelnuts, air pollutants, oncogenic viruses (the Human Papillomaviruses, the Epstein-Barr virus), poor oral hygiene, as well as inheritance (e.g., Fanconi anemia)^15^. By and large, these carcinogens damage DNA of the head and neck epithelium and cause accumulation of genetic aberrations leading to HNSCC.

Aside from several FDA-approved tissue agnostic precision medicines that may cover a small subset of HNSCC patients [i.e., infrequent *NTRK1/2/3*-rearrangments and *ROS-1-*rearrangement for larotrectinib and entrectinib, respectively], and an immune-hot subset of patients (~20–25% of patients) who respond to PD-1/PD-L1 inhibitors for reasons undefined, ~75–80% of HNSCC patients remain clinically inactionable by precision medicine.

As high as ~18% of HNSCC patient tumors harbor MAPK pathway mutations^7^. Core pathway components (*HRAS*, *BRAF*, *MAPK1*, *RPS6KA1*) are mutated in ~10.5% cases (54/512, TCGA-HNSCC cohort), while key scaffold proteins and negative regulators are mutated in ~4% and ~3% cases, respectively. Functional genomics and bioinformatics demonstrate that nearly half of the HNSCC-associated MAPK pathway mutations are activating or oncogenic in nature (Supplementary Fig. 1). Examples include *HRAS* p.G12/G13/Q61, *MAPK1* p.E81/E322, *MEK1* p.K57/E102, *MEK2* p.F57 and *BRAF* p.G466/D594/V600 mutations, all being recognized drivers for tumorigenesis. Some of which can cause oncogene addiction in cancer cells (where cancer cells are dependent on these oncogenic signaling for survival), thus can be potentially harnessed for therapy development. Yet, the clinical druggability of MAPK mutational events in HNSCC has remained unaddressed for a long time.

**Fig. 2:**
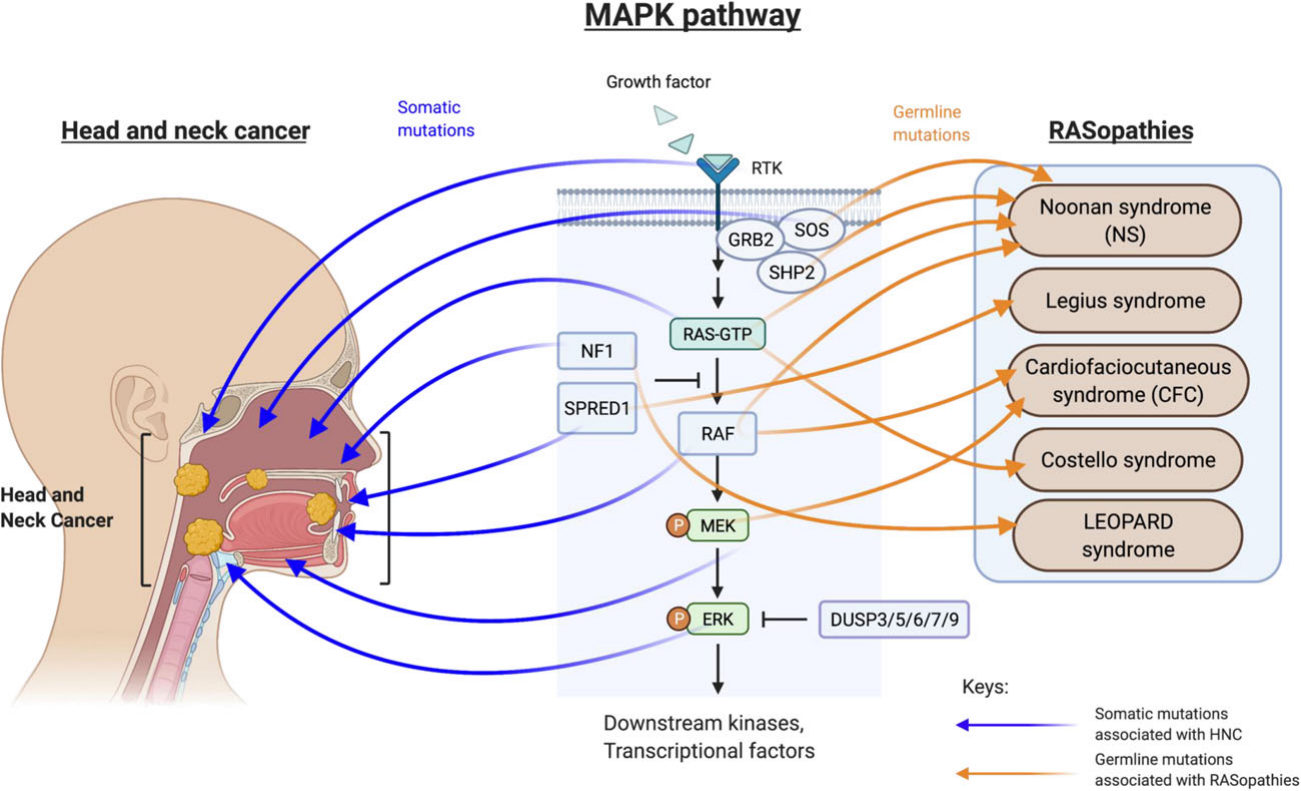
Components of the MAPK pathway are mutated in both HNSCC (at somatic level) and in various RASopathies at germline level. These RASopathies display head and neck deformities include the Noonan syndrome, Legius syndrome, cardiofaciocutaneous syndrome, and Costello syndrome.

For decades, the medical term RASopathies define genetic syndromes caused by various germline mutations of MAPK kinases or negative regulators. Individuals with RASopathies display hallmark craniofacial developmental and congenital abnormalities, implicating key roles of germline MAPK pathway mutations in regulating the development and growth of the head and neck region in human. Gain-of-function mutations of *BRAF* p.Q257R, *MEK1* p.Y130C and *MEK2* p.F57C are clinically associated with cardiofaciocutaneous (CFC) syndrome, *KRAS* p.T58I and *RAF1* p.S257P mutations with Noonan syndrome, *HRAS* p.G12S mutation with Costello syndrome, *NF1* mutation with LEOPARD syndrome, while loss-of-function mutations of the *SPRED1* negative regulators rendering activated MAPK signaling is associated with Legius syndrome^16^ (Fig. 2). Interestingly, some of these defects, but in a somatic manner, have been identified in HNSCC as well. Shared MAPK pathway aberrations in HNSCC and RASopathies are shown (Fig. 2). For instance, *HRAS* mutations, mutated in 6.6% HNSCC (but uncommon in other solid tumors), are also germline altered in Costello syndrome. The shared MAPK-mutated pathway components found in HNSCC and RASopathies raise attention not only for head and neck pathobiology understanding but also for therapeutic development against these mutations for HNSCC and likely RASopathies.

### Past Failures of MAPK/MEK kinase inhibitor trials in HNSCC

Prior to the genomic era, various preclinical studies suggested the potential therapeutic value of MAPK pathway inhibitors for HNSCC treatment^17–19^. It has been documented that HNSCC patients with high intratumor expressions of p-MAPK1/3 (p-ERK1/2) have poor survival^20^, and inhibition of p-ERK1/2 by MAPK pathway inhibitors often inhibited HNSCC cell growth in vitro^21–23^. However, when it came to clinical testing of MEK/MAPK inhibitors, all HNSCC clinical trials have failed with a general lack of efficacies in unstratified patients. These failed attempts with MEK/MAPK inhibitors are summarized in Table 1, which include MEK1/2 inhibitors AZD8330, selumetinib, cobimetinib, TAK-733, (or combinations) and the ERK1/2 inhibitor MK-8353. Specifically, no objective response was observed in HNSCC patients treated with AZD8330 (NCT00454090), selumetinib (NCT00085787), cobimetinib (NCT00467779), and TAK-733 (NCT00948467)^24–27^. Several combination treatments, including selumetinib with tremelimumab and/or MEDI4736 (NCT02586987), and cobimetinib with Atezolizumab (NCT03264066), have also been tested in HNSCC patients under pan-cancer clinical trial settings without promising outcomes. Similarly, HNSCC patients did not show any objective responses towards the ERK1/2 inhibitor, MK-8353 in a Phase I trial (NCT0135833)^28^. Basically, all trials ended at Phase I settings with no further studies thereafter. In fact, other than clinical failures in HNSCC, MEK1/2 inhibitors, such as CI-1040, was also demonstrated to be ineffective against solid tumors with trials halted at Phase II settings^29,30^.

**Table 1:** Failed clinical trials for MAPK pathway inhibitors in HNSCC patients.

Clinical trial ID	Clinical trial (tumor type; *N*)	Target(s)	Drug(s)	No. of HNSCC patients	HNSCC patient outcome	Outcomes of non-HNSCC patients	Ref
**NCT00454090**	Phase I (multiple solid tumors; *N* = 82)	MEK1/2	AZD8330	8	Not specified	Manageable toxicity at 20 mg BID (MTD); 1 melanoma case with partial response; 22 other patients with stable disease (>3mo).	^24^
**NCT00085787**	Phase I (multiple solid tumors; *N* = 57)	MEK1/2	Selumetinib	2	Not specified	19 patients had stable disease at the end of cycle 2 (≥ 5mo of stable disease in 9 patients).	^25^
**NCT02586987**	Phase I (multiple solid tumors; *N* = 58)	MEK1/2	Selumetinib [with MEDI4736; with MEDI4736 + Tremelimumab (anti-CTLA4 antibody)]	N.A.	N.A.	“Completed” but result unavailable.	N.A.
**NCT00467779**	Phase I (multiple solid tumors; *N* = 97)	MEK1/2	Cobimetinib	5	No objective responses reported	In melanoma patients, 1 complete and 6 partial responses.	^26^
**NCT03264066**	Phase I (multiple solid tumors; *N* = 88)	MEK1/2	Cobimetinib [with Atezolizumab (anti-PD-L1 antibody)]	N.A.	N.A.	“Completed” but result unavailable.	N.A.
**NCT00948467**	Phase I (multiple solid tumors; *N* = 51)	MEK1/2	TAK-733	1	No objective responses reported	2 partial responses (cutaneous melanoma).	^27^
**NCT01358331**	Phase I (multiple solid tumors; *N* = 25)	ERK1/2	MK-8353	1	Discontinued after 56 days of treatment (200 mg BID) due to progression	3 *BRAF* p.V600E-mutant melanoma patients out of 15 evaluable patients demonstrated partial responses.	^28^

Besides the lack of clinical efficacies for HNSCC, these early MEK/MAPK inhibitors appeared to cause dose-limiting toxicities in human. Early MEK1/2 inhibitor trials in pan-cancers were halted due to toxicities in patients. For instance, PD0325901, a second-generation MEK1/2 inhibitor with improved potency and bioavailability, had caused dose-dependent skin rash and dose-limiting gastrointestinal toxicity (diarrhea), hematologic, acute neurologic and ocular toxicities, and musculoskeletal adverse events, such as muscular weakness^31–33^. Similarly, pimasertib (MEK1/2 inhibitor) in combination with voxtalisib (mTOR/PI3K inhibitor), also elicited ocular and cardiac toxicities (NCT01390818); CC-90003 (ERK1/2 inhibitor) caused grade 1-3 neurotoxicity (NCT02313012), and GDC-0994 (ERK1/2 inhibitor) in combination with cobimetinib (MEK1/2 inhibitor) caused grade 3 dose-limiting toxicities including myocardial infarction and rash (NCT02457793) in advanced cancer patients^34–36^. Thus, there were cautions for the use of MEK/MAPK inhibitors in various cancers, especially there seems a general lack of clinical benefits in patients.

### Drugging HNSCC with MAPK pathway inhibitors: potency and precision

As in other cancers, the reasons behind the general lack of clinical efficacies of early MEK/MAPK inhibitor trials in HNSCC remains unclear, but likely complex. These may include drug resistance mechanisms, drug potency issues, and most importantly, potential wrong ways of drugging this pathway in HNSCC^37^.

First, various MAPK pathway inhibitors were known to cause feedback, feed-forward, cross-talk signaling that help cancer cells re-gaining MAPK activation despite initial perturbation by the inhibitors, resulting in acquired resistance to these inhibitors in cancer cells. Studies have identified *NRAS* or *MEK* activating mutations, *RAF* amplification, RAF heterodimerization, *BRAF* alternative splicing, loss of *NF1*, etc., as causes for acquired resistance to MAPK inhibitors^37^. Recent strategies, aiming at double-striking 2 nodes of the MAPK pathway have gained much attention as a likely and feasible way to interrupt multiple feedbacks in many cancers, though the efficacies of these strategies have not been clinically evaluated in HNSCC yet. Interestingly, a preclinical study revealed that acquired resistance against selumetinib (MEK inhibitor) was attributed by FGFR3-mediated MAPK reactivation in HNSCC, suggesting combined FGFR3/MAPK to resist the development of selumetinib resistance in HNSCC^38^.

Second, insufficient potencies of older generations of MAPK inhibitors may underlie their clinical failures in HNSCC in the past^39^. Indeed, in recent years, new classes of MEK inhibitors have been developed with improved potencies, and have shown some clinical promises in HNSCC, and other cancers. Of all, trametinib is one promising MEK1/2 inhibitor for HNSCC. In 2013, a Phase II window-of-opportunity trial with neoadjuvant trametinib demonstrated marked median tumor size reduction (46%) in all patients with reduction and 11 patients with partial responses among 17 evaluable patients (Stage II-IV oral cancer). Of note, 5 patients showing reduced intratumoral p-ERK1/2 levels post-treatment were all found to be clinical responders by tumor size or tumor metabolic criteria^40^. The promising trial results implicate that potent MEK/MAPK inhibitors may be required for effective treatment of HNSCC.

Third, which is the most important question to ask is: whether we have been drugging this pathway wrongly in the past among HNSCC patients? Would past failures be contributed by the unstratified patient pools (one-for-all), as we did not have ways to identify which patients are genetically-condemned with MAPK pathway dependencies? ***Now, with the understanding of the genomic features of HNSCC patients, shall we drug the MAPK-condemned patients differently and be able to see good clinical outcomes in those patients?***

In fact, promising results from a recent RAS inhibitor trial with tipifarnib sheds light for effective precision drugging of *HRAS*-mutated HNSCC patients (NCT03719690) and have resulted in fast-track drug review by the FDA (detailed discussion below). Contrasting with previous failures of MEK/MAPK inhibitors for HNSCC, this FTI is paving its way as a likely first precision medicine for a noticeable subset of HNSCC patients (~6% cases). Details on the clinical successes of tipifarnib and additional Ras inhibitors for HNSCC are discussed below. Furthermore, new clinical findings including many exceptional responder studies, and recent -omics studies do suggest new avenues for precision drugging of HNSCC patients bearing either germline or somatic MAPK aberrations, highlighting the need for future investigations in the clinic.

#### Tipifarnib Reveals the Need for Precision Drugging in HNSCC

RAS proteins are small GTPases activated upon GTP binding to stimulate downstream RAF/MEK/MAPK signaling for cell growth. As membrane localization of RAS proteins is required for their activation, pharmacologic inhibition of RAS post-translational modifications, such as C-terminal farnesylation (by inhibiting the enzyme farnesyl transferase that transfers farnesyl group to the C-terminal of RAS), could potentially inactivate RAS. Till now, four farnesyl transferase inhibitors (FTIs), namely L-778,123, BMS-214662, tipifarnib, and lonafarnib have been clinically evaluated in various cancers, including HNSCC (Table 2). Their effects in HNSCC patients are discussed below.

**Table 2:** Failure of early non-precision clinical trials for RAS inhibitors or farnesyl transferase inhibitors (FTIs) involving HNSCC patients.

Clinical trial ID	Clinical trial (tumor type; N)	Target(s)	Drug(s)	No. of HNSCC patients	HNSCC patient outcome	Outcomes of non-HNSCC patients	Ref
**N.A**.	Phase I (HNSCC and NSCLC; *N* = 9)	FT/GGTase-I	L-778,123 [with standard radiotherapy]	3	2 had complete clinical respones with no evidence of disease at follow-up examinations, but MTD of th L-778,123/radiation combination was not well-defined.	Of the 4 evaluable NSCLC patients, 3 had a complete response and 1 had a partial response.	^41^
**NCT00003430**	Phase I (HNSCC and unspecified solid tumors; *N* = N.A.)	FT/GGTase-I	L-778,123	N.A.	“Completed” but results unavailable.	N.A.	N.A.
**NCT00006242**	Phase I (multiple solid tumors; *N* = 44)	FT	BMS-214662	2	No objective responses reported.	Antitumor activity was observed in 1 h I.V. infusion in patients. A pancreatic cancer patient has prolonged stable disease for more than five years under BMS-214662 treatment; A NSCLC patient has 40% size reduction in liver metastasis and complete shrinkage in one of three brain metastases.	^42^
**NCT00005973**	Phase I (multiple solid tumors; *N* = 30)	FT	BMS-214662	6	No objective responses reported.	A minor response in a NSCLC patient; A weekly 1 h infusion did not provide sustained and substantial enough FTase inhibition for apoptosis.	^43^
**NCT00038584**	Phase Ib (HNSCC; *N* = 37)	FT	Lonafarnib	37	“Clinical responses” were seen at all lonafarnib dose levels (oral 100 mg twice daily (b.i.d.), 200 mg b.i.d., or 300 mg b.i.d. for 8 to 14 days before surgical resection), with marked tumor reduction in 4 of 22 evaluable patients. 3 of 17 treated patients demonstrated partial responses.	N.A.	^44^
**NCT00073450**	Phase II (HNSCC; *N* = 15)	FT	Lonafarnib	15	7 patients (47%) had stable disease; Well-tolerated but no objective responses observed in the first 15 patients enrolled, and the study is terminated to further accrual.	N.A.	^45^
**NCT00102635**	Phase I (HNSCC; *N* = N.A.)	FT	Lonafarnib [with 4-HPR]	N.A.	Terminated due to slow accrual.	N.A.	N.A.

L-778,123: a Phase I trial aimed to determine L-778,123’s maximal tolerated dose (MTD) in combination with radiotherapy in 9 advanced cancer patients (3 with stage IV HNSCC, 6 with NSCLC)^41^. Among the 3 HNSCC patients, despite one patient falling short of complete treatment due to toxicity, the remaining 2 patients have demonstrated complete responses with no evidence-of-disease (NOD), at follow-up examinations. Ras mutational statuses were not clearly reported. Despite complete responses observed, the MTD of the L-778,123/radiation combination could not be defined^41^. It was also noted that another Phase I clinical trial for L-778,123 was conducted by the Memorial Sloan Kettering Cancer Center and National Cancer Institute (NCT00003430) for patients with recurrent or refractory solid tumors including HNSCC, but no trial result was published.

BMS-214662: a potent FTI of the 1,4-benzodiazepine class with IC_50_ value of low nM range in vitro. Two HNSCC patients involved in a mixed solid tumor Phase I trial, were treated with BMS-214662 and did not show objective responses, while another Phase I BMS-214662 trial with HNSCC patients did not report patient outcomes in detail^42,43^.

Lonafarnib (SCH66336): a tricyclic peptidomimetic inhibitor being one of the first FTIs underwent human clinical trials for cancer, including both Phase I and II trials involving HNSCC patients. An early Phase Ib trial result released in a conference proceeding reported clinical responses at all lonafarnib doses tested in patients prior surgery (100 to 300 mg twice daily), with marked tumor size reduction in 4/22 evaluable patients^44^. A later Phase II lonafarnib trial in chemo-refractory advanced HNSCC showed that among 15 patients who previously failed platinum-based therapies, 7 had stable diseases in a minimum of 3 cycles of treatment, while 1 patient was stable for 8 cycles of treatment for 220 days total. Further, lonafarnib was well-tolerated with no grade 3 or 4 hematologic toxicities^45^. Among these advanced HNSCC patients, these stable disease cases with lonafarnib suggested potential therapeutic efficacy of FTIs for HNSCC, which was later shown by tipifarnib. Interestingly, lonafarnib was recently approved by the FDA (Nov, 2020) for the treatment of a form of premature aging disease called Hutchinson-Gilford progeria syndrome, and progeroid laminopathies to reduce their risk of death by preventing the build-up of “defective progerin or progerin-like protein” that causes accelerated heart failure.

Tipifarnib: a potent FTI (Supplementary Fig. 2), with IC_50_ values of 0.86 nM and 7.9 nM against lamin B and K-RasB peptide substrates in vitro^46^. With the realization that *HRAS* mutations can cause oncogene addiction and activated MAPK signaling in cancer, tipifarnib trials have sought to investigate its effectiveness in pan-cancer patients with and without *HRAS* mutations. In a recent phase II trial (NCT02383927), tipifarnib demonstrated a 53% overall response rate (13/23 patients) among HNSCC patients^47^. Furthermore, significant clinical activity was noted in patients with recurrent and metastatic HNSCC harboring *HRAS* mutation with high variant allele frequency (VAF) of >20%. Eight out of 15 HNSCC patients meeting such a criterion had partial responses, while additional 5 demonstrated stable diseases (53% response rate) with tipifarnib^47^. Tipifarnib also demonstrated modest clinical activity in patients with recurrent, metastatic *HRAS*-mutant salivary gland cancer, and urothelial carcinoma. Among 12 evaluable *HRAS*-mutant recurrent metastatic salivary gland cancer (SGC) patients, one demonstrated partial response and 7 had stable disease with median duration of response of 9 months^48^. In this small SGC cohort, the kind of *HRAS* variants and allele frequency did not correspond to clinical outcome with tipifarnib. Nevertheless, these promising clinical findings provide strong evidences for the use of *HRAS* mutations in HNSCC as predictive biomarkers for tipifarnib sensitivity. In general, tipifarnib was well-tolerated, with fatigue, myelosuppression, nausea, and vomiting being the most common adverse events (all grades) observed^47^. Currently, the FDA has granted fast-track designation to tipifarnib for *HRAS*-mutant HNSCC whose disease progressed on platinum therapy^49^. An ongoing international Phase II trial is specifically evaluating tipifarnib efficacy in HNSCC patients with high *HRAS*-mutant VAF (NCT03719690). It is very likely that *HRAS*-mutant HNSCC may represent the first precision medicine specific for HNSCC.

Alongside, studies are ongoing to start tackling potential tipifarnib-resistance in HNSCC. Using *HRAS*-mutant HNSCC patient-derived xenografts (PDXs), tipifarnib-resistance was identified to involve aberrant apoptosis and angiogenesis^50^. Nevertheless, their involvement and clinical relevance in HNSCC remains unknown. A recent CRISPR-screen has already identified potential combination strategy of tipifarnib with autophagy inhibitors to prepare for even more effective tipifarnib treatment for *HRAS*-mutant HNSCC^51^.

#### Drugging HNSCC with KRAS Germline Variants by Cetuximab Addition

As tipifarnib demonstrates good clinical efficacies in *HRAS*-mutated HNSCC, salivary gland carcinoma, and urothelial carcinoma^48,52,53^, other RAS genes have gained attention for precision medicine development. New RAS inhibitors, AMG-510 and MRTX849, have been recently developed and showed clinical promises in patients with *KRAS* p.G12C-mutated solid tumors (NCT03600883, NCT04330664)^54^. In fact, AMG-510 has recently been FDA-approved for the treatment of *KRAS* p.G12C-mutated locally advanced or metastatic NSCLC (May, 2021). Unlike *HRAS*, the mutation rate of *KRAS* is relatively low in HNSCC (1/512; 0.2%). Yet, we recently reported that *KRAS* mutation rate can found in ~3.5% of advanced HNSCC^55^. In metastatic larynx cancer, *KRAS* mutation may account for ~6% cases^56^. Furthermore, *KRAS* mutations appear to be a poor prognostic biomarker for advanced HNSCC, with shorter disease-free survival in *KRAS*-mutant vs. WT patients^55^.

Among all new KRAS inhibitors under clinical evaluation, only AMG-510, MRTX849, and LY3499446 have entered phase II settings^57^. Yet, the low rate of *KRAS* somatic mutations in HNSCC presents a challenge for clinical assessment of KRAS inhibitors with *KRAS*-status stratification.

Interestingly, Weidhaas et al. recently performed a secondary analysis of a randomized phase III HNSCC trial and found that patients with an oncogenic germline *KRAS* variant (a let-7 microRNA-binding site polymorphism in the 3’ untranslated region of KRAS) have significantly better clinical outcomes with cetuximab (EGFR monoclonal antibody) addition to radiotherapy plus cisplatin regimen^58^. Furthermore, these germline *KRAS*-variant HNSCC patients were found to have increased plasma TGF-β1, potentially contributing to immunosuppression in these patients. Thus, targeting with cetuximab may help these patients overcome TGF-β1–induced immunosuppression. In fact, this very same *KRAS* germline variant also predicts good clinical response to cetuximab monotherapy in otherwise somatic *KRAS*-WT metastatic colon cancer patients^59^. Thus, *KRAS* germline variant guiding combination therapy with cetuximab, and potentially radiotherapy/cisplatin regimen should be further evaluated in larger prospective trial in HNSCC. The main side effects reported in the trial were skin reaction, some with grade 3 to 4 mucositis^58^.

#### Exceptional Erlotinib responses for MAPK1-mutant HNSCC

About 5% of HNSCC patients harbor somatic mutations of *MAPK1* (*ERK2*) gene^12^. In 2015, Van Allen and Lui et al. reported the first HNSCC exceptional responder (with Stage III advanced oral cancer) who exhibited a complete response (>30 months) to a 13-day erlotinib treatment^60^. This clinical finding is highly unexpected given another EGFR targeting agent, cetuximab often demonstrates moderate activities in HNSCC patients in general. Whole-exome characterization of pre-treatment biopsy showed no *EGFR* aberrations, but the presence of a somatic *MAPK1* p.E322K mutation using an EGFR-network bioinformatics approach. This mutation was then functionally characterized to be a potent driver for constitutive ERK activation and HNSCC cell growth^12^. Subsequent investigation revealed its ability to drive robust EGFR hyperactivation by enhancing autocrine amphiregulin release from HNSCC cells, thus hyperactivating EGFR signaling^61^, and rendering hypersensitivity to an EGFR kinase inhibitor, erlotinib. The study was later extended to additional *MAPK1* mutations in HNSCC, and led to the finding that *MAPK1* p.D321N (also hyperactivates MAPK and p-EGFR) could also confer heightened sensitivity to erlotinib in HNSCC in vivo, while *MAPK1* p.R135K mutation (moderately activating p-EGFR) conferred moderate level of erlotinib sensitivity^12^. Importantly, both *MAPK1* p.D321N and p.R135K mutations exist in recurrent HNSCC in Asia^12^. These results suggested that MAPK (or ERK) activities could be associated with erlotinib responses in HNSCC. In fact, results from the randomized clinical trial (NCT00779389) involving the first HNSCC exceptional responder eventually concluded that baseline tumoral p-MAPK (p-ERK) levels were inversely correlated with tumor size post-erlotinib treatment, showing that MAPK activation status is likely an indicator of EGFR-addiction in HNSCC, and thus predictive of clinical responses to erlotinib in HNSCC patients^62^. Overall, the clinical trial reported brief exposure to erlotinib was well-tolerated in HNSCC, with acneiform rash and diarrhea being major side effects that are commonly observed with EGFR inhibitors^61^.

Interestingly, based on current genomic findings, US TCGA-HNSCC patients with primary tumors universally harbor *MAPK1* p.E322K mutations, while an Asian primary/recurrent HNSCC cohort showed a wide spectrum of p.E322K/D321N/R135 mutations^12^. As EGFR kinase inhibitors (e.g., erlotinib, gefitinib) are known to be clinically safe for treatment of lung cancer, these clinical and findings provide important scientific evidences supportive of precision trials for *MAPK1*-mutated, yet EGFR-addicted HNSCC.

#### BRAF p.V600E-mutated Ameloblastoma are Exceptionally Sensitive to BRAF/MEK Inhibitors

Ameloblastoma is a rare head and neck tumor (1.79/10,000,000 persons/year) found in the jaw area near the molar^63^. Though rare, high rates of *BRAF* p.V600E hotspot mutation (33.3–82%) have enticed growing interest for BRAF-targeting, as ameloblastoma often requires extensive facial surgeries, compromising quality-of-life, yet, frequently recurs^64–68^.

Five exceptional responder reports have been documented with dabrafenib or vemurafenib monotherapies, and dabrafenib/trametinib combination (Table 3). Kaye et al. first reported an African American ameloblastoma patient with recurrent metastases bearing somatic *BRAF* p.V600E mutation (detected by mutant-specific antibody with immunohistochemistry), treated with compassionate dabrafenib (150 mg twice daily) plus trametinib (2 mg once daily) and exhibited immediate marked tumor reduction at day 4, followed by disappearance of lung metastases and shrinkage of head and neck lesions at week 8, and persistent antitumor responses even at 20 weeks^69^. This combination was well-tolerated clinically. Another remarkable clinical responses to this combination was also observed in a 26-year old recurrent metastatic ameloblastoma patient showing complete dissolution of lung metastasis and primary tumor at 30 weeks after treatment (NCT02534649)^70^. The NCT02367859 trial also reported a stable disease for a *BRAF* p.V600E-mutated ameloblastoma patient. These cumulative responder cases strongly suggest the potential therapeutic benefits of BRAF/MEK inhibition in *BRAF* p.V600E-mutated ameloblastoma (note: such combination is FDA-approved for *BRAF* p.V600E-mutated thyroid cancer and melanoma). Besides, two other recurrent *BRAF* p.V600E-mutated ameloblastoma patients have also responded dramatically to dabrafenib monotherapy. These include an 83-year-old patient treated with low dose dabrafenib (75 mg twice daily) showing 75% tumor size reduction at 12 months^71^, and another recurrent patient responding to dabrafenib with >90% tumor shrinkage^72^. Lastly, besides dabrafenib, the use of vemurafenib has also caused persistent and marked tumor shrinkage (>60% for 11 months) in a recurrent *BRAF* p.V600E-mutated ameloblastoma patient^73^. Common reported side effects for these BRAFi, MEKi and their combinations include asthenia, rash, arthralgia, nausea, pyrexia, fatigue and headache, etc., and they, in general, showed good toxicity profiles in patients^70^. Thus, *BRAF* p.V600E-mutated ameloblastoma could be pharmacologically vulnerable for precision BRAF-targeting.

**Table 3:** Exceptional responders to BRAF and/or MEK inhibitors among patients with *BRAF* p.V600E-mutated recurrent/metastatic ameloblastoma.

Cancer type	Tumor stage	Target(s)	Drug(s)	Response by RECIST and PERCIST criteria	Duration of treatment	Details on tumor volume reduction	Ref
***BRAF*p.V600E Ameloblastoma**	Metastatic	MEK1/2 and BRAF	Dabrafenib and Trametinib	Partial response	20 weeks	Visible tumor size reduction shown in pre- and post-treatment PET-CT images 8 weeks after treatment onset (reduction percentage unreported).	^69^
	Metastatic	MEK1/2 and BRAF	Dabrafenib and Trametinib	Complete response	30 weeks	Complete response ongoing 30 weeks after onset of treatment.	^70^
	Recurrent	BRAF	Dabrafenib	Partial response	12 months	75% tumor size reduction at 8 months with 50% standard dose of dabrafenib for metastatic melanoma, and durable response continued at 12 months.	^71^
	Recurrent	BRAF	Dabrafenib	Partial response	73 days	No change in tumor size but over 90% tumor volume reduction characterized by degeneration and squamous differentiation of the inner parts of tumors.	^72^
	Recurrent	BRAF	Vemurafenib	Partial response	11 months	Reduction of lesion size from 24 × 21 × 19 mm to 18 × 13 × 14 mm 11 months after treatment initiation.	^73^

#### Remarkable clinical responses to MAPK inhibitors in BRAF-mutated HNSCC

Unlike ameloblastoma, *BRAF* mutations only occur in ~1.8% of HNSCC, and the clinical responsiveness of *BRAF*-mutated HNSCC patients to BRAF inhibition remains under-explored. Yet, a recent Phase I trial (NCT01781429) did report a *BRAF* p.G469A-mutated head and neck cancer patient with partial response to ulixertinib (an ERK1/2 inhibitor) per RECIST criteria (> 30% tumor size reduction)^74^. Though such a dramatic ulixertinib response only lasted for 4.9 months in this patient, a little fall short of the NCI’s criteria for exceptional responders (6-months), this report does provide clinical evidences supportive of future ulixertinib trials in relation to *BRAF*-mutational status for this relatively safe drug. In the trial, the most common treatment-related adverse effects were rash, diarrhea, fatigue, and nausea, with no grade 4 or 5 treatment-related adverse effects reported.

#### MAPK mutations modulate ErbB3 activation in HNSCC: potential therapeutic consideration

ERBB3 is a new target for HNSCC. Its activation level (p-ErbB3) is significantly associated with poorer HNSCC patient survival^7^. Various ErbB3 inhibitors are under development. Among which, CDX-3379, an anti-ErbB3 monoclonal antibody, has demonstrated antitumor activity resulting in tumor shrinkage in 42% of HNSCC patients, with grade 1 to 2 diarrhea, fatigue, and acneiform dermatitis being the most often treatment-related toxicities^75^.

A recent study showed that multiple MAPK pathway mutations found in HNSCC patient tumors, including *BRAF* p.V600E, *HRAS* p.G12V, *MEK1* p.K57N, *MEK2* p.F57L, *MAPK1* p.E322K, *MAPK1* p.D321N, *ARAF* p.S214F, *ARAF* p.P508L, as well as wildtype *A/BRAF* genes could significantly suppress p-ErbB3 levels in HNSCC cells^7^. Moreover, pharmacologic inhibition of MAPK1/3 activity by GDC-0994 has resulted in p-ErbB3 activation in MAPK-mutant HNSCC primary cultures and cell models, but not in WT counterparts, indicating that MAPK activating mutations can modulate p-ErbB3 levels in HNSCC. This was further supported by findings in HNSCC patient tumors with high allele frequencies of *HRAS* p.G12S and *MAPK1* p.D321N mutations (>30-40% allele frequency) expressing low levels of p-ErbB3. Thus, MAPK mutational status or ERK activities can modulate ErbB3 activation level in HNSCC, suggesting the need for precautions when using ErbB3 inhibitors in MAPK-mutant HNSCC patients. Further clinical studies should be conducted to determine if perhaps, ErbB3 inhibitors may/should be avoided in MAPK-mutant HNSCC patients.

#### MAPK-mutant HNSCC are CD8^+^T cell-inflamed: implications for T cell-based immunotherapies

HPV(+)HNSCC patients are known to respond well to T cell-based immunotherapies, including PD1 inhibitors pembrolizumab and nivolumab^76–79^. This is at least partially contributed by elevated immune infiltrates in HPV(+)HNSCC tumors^76–82^. Numerous PD1 inhibitor trials confirmed that HPV(+)HNSCC patients have reduced hazard ratios for death (HR = 0.82, *p* = 0.0316) when compared to standard treatment^83^. For recurrent and metastatic HNSCC, HPV(+)HNSCC patients demonstrated a better objective response rate to durvalumab than HPV(-)HNSCC patients (29.4% vs 10.8%, with median OS = 10.2 months 5.0 months)^84^. However, HPV status was not associated with clinical responses to nivolumab in the CheckMate 141 study^85^. Recent bioinformatic analysis of HNSCC patient tumors showed that MAPK-mutant HNSCC tumors, irrespective of HPV status, also have elevated CD8^+^T cell and dendritic cell infiltrations, together with immune-active cytolytic and interferon-gamma signatures in situ, suggestive of an active cytolytic CD8^+^T-inflamed status in these tumors. Interestingly, this CD8^+^T-inflamed and cytolytic feature was not shared by HNSCC tumors bearing PI3K, NOTCH, JAK/STAT, WNT, NF-κB, or TGFβ/Smad pathway mutations^7^. The study further demonstrated that ectopic expression of MAPK pathway mutations (mouse *HRAS* p.G12V, mouse *MAPK1* p.D319N and p.E320K), but not respective WT-counterparts, could directly shape the HNSCC immune tumor microenvironment by attracting CD8^+^T cell infiltration in immunocompetent mouse allografts^7^. Whether such MAPK mutation-driven CD8^+^T cell infiltration was caused by activated MAPK signaling or the associated neoantigens warrants further investigations. It also remains to be determined if these CD8^+^T cells are clonal or not. Using TCGA-HNSCC dataset, Lyn et al. also showed immune-enhancing signatures associated with *HRAS* mutation, including elevated CD8^+^ markers and *HLA* gene expressions, high cytolytic activity, and immune scores^86^.

Since CD8^+^T cells are effective antitumor immune cells, its markedly increased level in MAPK-mutant HNSCC tumors may tie to PD1 inhibitor response. In fact, subsequent retrospective analysis of anti-PD1 immunotherapy cohorts did reveal that MAPK pathway mutations could also predict anti-PD1 immunotherapy outcome in advanced and metastatic oral cancer patients^7^, with 2-3 times longer median survival than WT patients^7^. Thus, such a highly immunoactivated status of MAPK-mutant HNSCC tumors may potentially outweigh the oncogenic effects of these activating mutations, and constitute better clinical outcome to ICI in HNSCC patients. Large prospective ICI clinical investigation with known MAPK-mutation status should be warranted to determine the clinical efficacy of ICI therapy in HNSCC patients for precision immunotherapy.

#### Resistance of MAPK-mutated HNSCC?

Activation of the RAS/RAF/MEK/ERK signaling is known to contribute to, at least in part, resistance to several therapies in HNSCC. These include resistance to cisplatin^87^, EGFR-monotherapy (cetuximab) likely through cross-talk signaling with the PI3K/AKT pathway^5,88^, EGFR/HER-3 combination therapy^89^, PI3K inhibitor (BYL-719) via mTOR activation^90^, as well as new experimental agents such as CX-4945 (a Protein Kinase CK2 inhibitor^91^). As for resistance to radiotherapy, a German oropharyngeal cancer study (*N* = 124) showed that elevated p-Erk1/2 expression levels was associated with poor clinical outcomes, suggesting the potential involvement of ERK activation in HNSCC radioresistance^92^.

Future clinical trials are much anticipated for MAPK pathway-mutated HNSCC as discussed above due to the reported exceptional responders and good responders, and it is likely that resistance may also arise as in other precision medicines for many other cancers. Though resistance to “specific precision medicine treatments” of MAPK-mutated HNSCC is yet-to-be unfolded, researchers just begin to report general resistance to Cetuximab, an FDA-approved EGFR targeting antibody for HNSCC, in relation to *HRAS* mutations. In 2014, Rampias et al. reported an association of *HRAS* mutational status with de novo resistance to cetuximab-based chemotherapy (*P* = 0.046) in a small HNSCC cohort (7 *HRAS*-mutated vs 48 wildtype patients), suggestive of *HRAS*-mutant-related RAS/Erk activation for cetuximab resistance in HNSCC^5^. Though we cannot predict how such a wide array of HNSSC-associated MAPK pathway mutations may alter sensitivity and resistance to various agents, lessons from *BRAF* p.V600E-mutated melanomas and thyroid cancers do alert us of such possibility of resistance to long-term monotherapy treatment through reactivation of MEK/ERK signaling (and some other mechanisms including constitutive activation of EGFR and PI3K, etc.)^93^, which can be co-targeted by BRAF-MEK inhibitor combinations (e.g., encoragenib-binimetinib; dabrafenib-trametinib, etc) as approved recently by the FDA for other cancers.

## Conclusions

**Fig. 3:**
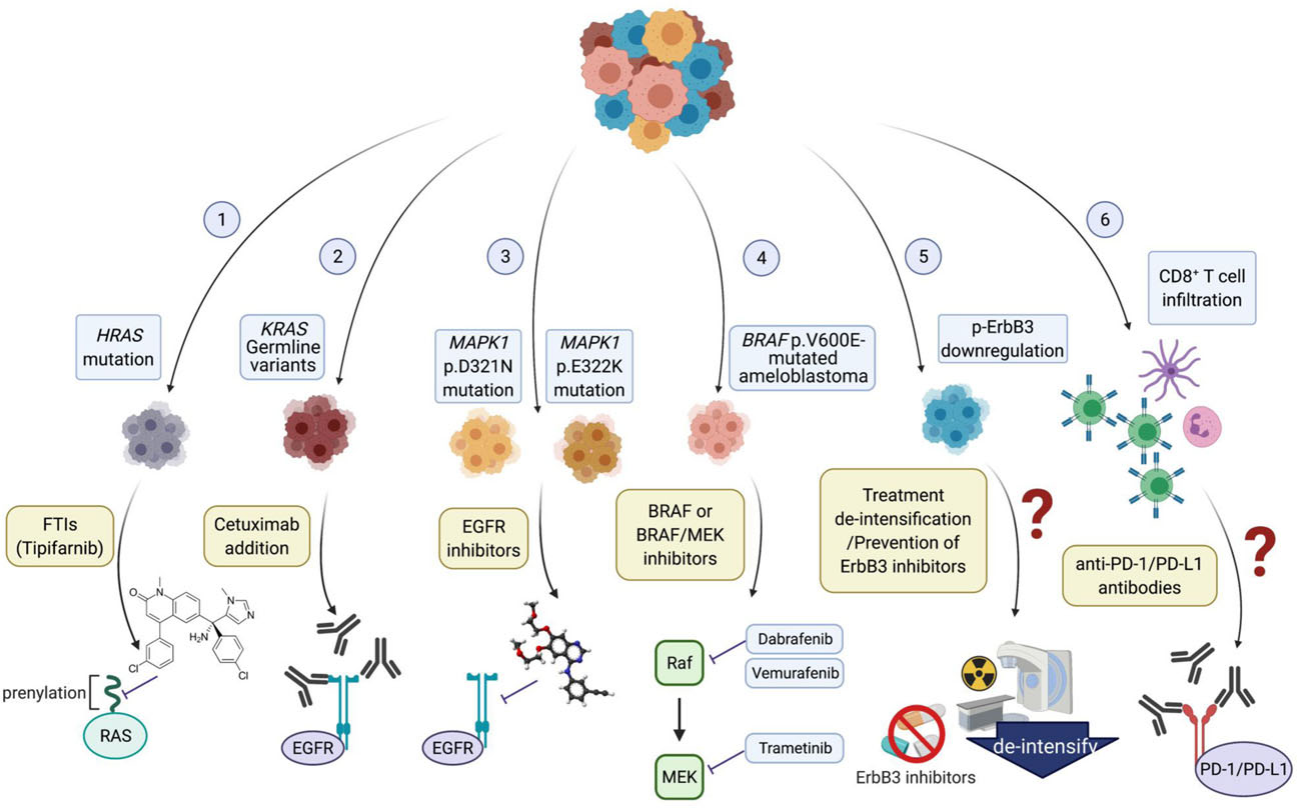
Proposed precision treatment strategies for MAPK pathway-mutated head and neck cancer. (1) FTIs, particularly tipifarnib, is currently under FDA fast track designation for *HRAS*-mutant HNSCC treatment; (2) HNSCC with *KRAS* germline variants are druggable with cetuximab addition; (3) MAPK1 mutations are targetable by EGFR inhibitors; (4) BRAF p.V600E-mutated ameloblastoma are exceptionally sensitive to and could be subject to BRAF monotherapy or BRAF/MEK combination therapy; (5) Treatment de-intensification or potentially “not-to-use” of ErbB3 inhibitors may need to be considered in MAPK-mutatedp-ErbB3 downregulated HNSCC. (6) CD8+ T cell infiltration in MAPK-mutant HNSCC may constitute better clinical outcome to anti-PD-1/PD-L1 inhibitor treatment.

In conclusion, based on recent clinical and omics findings, MAPK pathway mutations can be drug-sensitizing in HNSCC. Although the evolutionary or clonal nature of MAPK pathway mutated HNSCC is still unclear but definitely of interest for further investigations, this HNSCC subset should be given more attention for therapy development. Potential precision strategies may include EGFR kinase inhibitors, monoclonal EGFR antibody cetuximab, BRAF inhibitors such as vemurafenib or darafenib, newer generations of MAPK/MEK inhibitors, and CD8^+^T cell-based immunotherapies (Fig. 3). Somatic and germline mutation-based prospective trial design may help identifying new effective precision therapies for this previously undruggable MAPK pathway in HNSCC. For practical reasons due to the genetic heterogeneity of HNSCC tumors, if future MAPK precision clinical trial results are promising, it may be useful to perform a panel of MAPK pathway genes to facilitate future precision medicine implementations in the clinic.

### Reporting Summary

Further information on research design is available in the Nature Research Reporting Summary linked to this article.

## Supplementary information

Supplementary Information

Reporting Summary

## Data Availability

No datasets were generated or analyzed during the current study.
