# Supplementary material for: Precision drugging of the MAPK pathway in head and neck cancer
Source: NPJ Genom Med. 2022 Mar 16;7:20. doi: 10.1038/s41525-022-00293-1 (PMC8927572; doi:10.1038/s41525-022-00293-1)
Supplement: Supplementary file 1 — Supplementary Information [file 41525_2022_293_MOESM1_ESM.pdf]

Supplementary Information

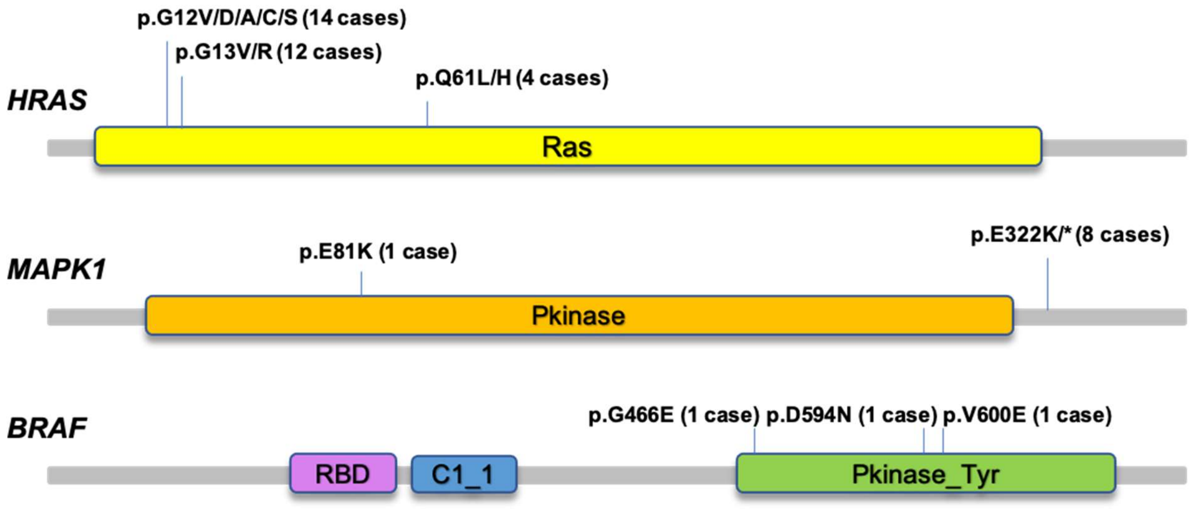

Supplementary Figure 1: Well-known activating MAPK pathway mutations found in TCGA HNSCC cohort. *Abbreviation:* Ras: Ras family (5 - 164), Pkinase: Protein kinase domain (25 - 313), RBD: Raf-like Ras-binding domain (156 - 227), C1_1: Phorbol esters/diacylglycerol binding domain (C1 domain) (235 - 280), Pkinase_Tyr: Protein tyrosine kinase (458 - 712).

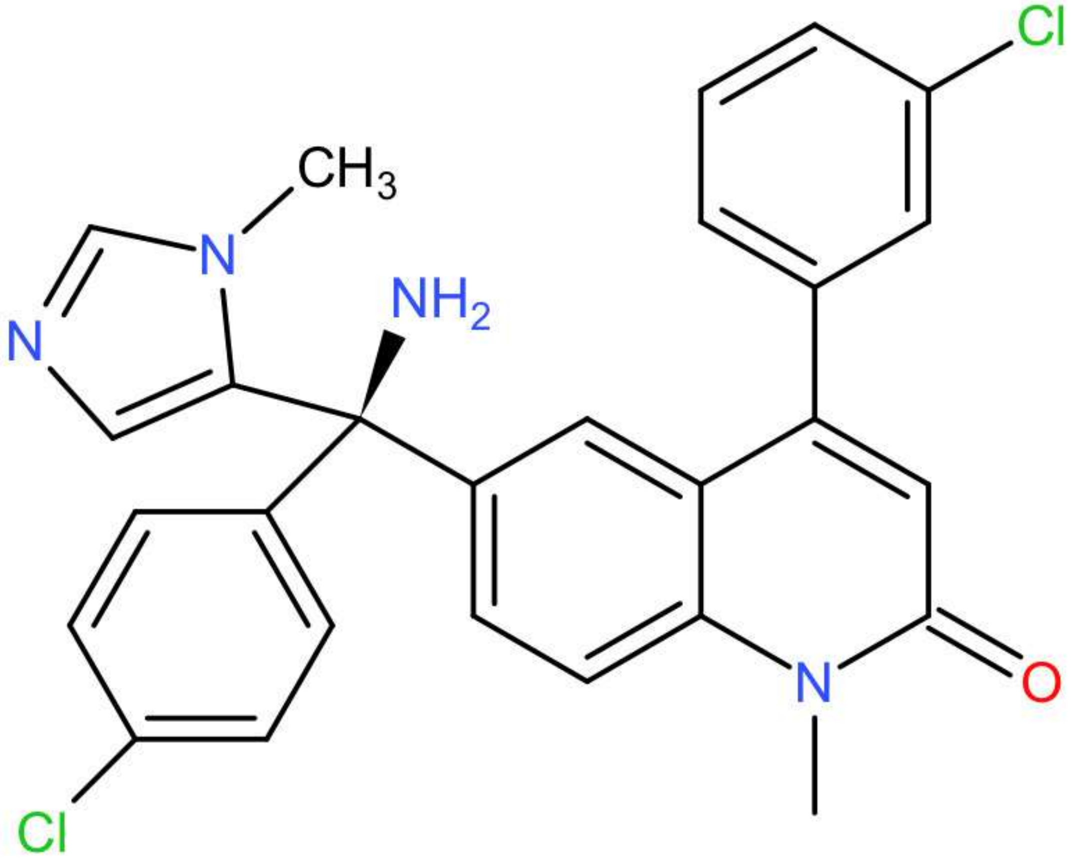

Supplementary Figure 2: Structure of tipifarnib, a farnesyltransferase inhibitor (FTI).
